# Soil iron modulates beneficial maize microbiome feedbacks in rotations with wheat

**DOI:** 10.1186/s40168-026-02493-8

**Published:** 2026-08-04

**Authors:** Jan Waelchli, Henry Janse van Rensburg, Katja Stengele, Viola D’Adda, Selma Cadot, Veronica Caggìa, Valentin Gfeller, Klaus Schlaeppi

**Affiliations:** 1 Department of Environmental Sciences, University of Basel, Basel, Switzerland; 2 Division of Agroecology and Environment, Agroscope, Zurich, Switzerland; 3 Institute of Plant Sciences, University of Bern, Bern, Switzerland; 4 Plant Breeding, Department of Crop Sciences, Research Institute of Organic Agriculture (FiBL), Frick, Switzerland

**Keywords:** Plant soil feedbacks, Crop rotations, Root exudates, Plant-available nutrients, Soil heterogeneity

## Abstract

**Background:**

Plants change their surrounding soil microbiome by root exudates and these conditioned microbiomes impact the performance of the present as well as the next plant generation as for example in crop rotations. The big challenge is that such ‘microbiome feedbacks’ are highly context-dependent, i.e. they vary in strength and direction dependent on the local soil environment – of which the driving factor(s) remain unknown. Including maize in crop rotations involves benzoxazinoids (BXs), which are exuded from roots and alter the soil microbiome, which in turn affects growth and defence of the following crop.

**Results:**

Here, we grew wild-type and BX-depleted maize in the field to differentially condition their soil microbiome and we found varying feedbacks on wheat performance dependent on the local physicochemical soil parameters. Using multivariate, correlation and modelling approaches and including additional data from two previous field experiments, we identified plant-available (PA) iron to be associated with BX-dependent microbiome feedbacks on wheat. The BX-conditioned soil microbiome caused wheat to grow taller at low levels of soil PA-iron but smaller at high levels. This finding was generalized by testing these maize microbiome feedbacks on the model plant *Arabidopsis thaliana* using soil batches containing different levels of iron. Consistent with wheat, a significant inverse relationship between soil PA-iron levels and plant growth was found. This relationship was experimentally validated with *Arabidopsis thaliana* grown at low levels of soil iron where iron supplementation abolished the beneficial feedback of the BX-conditioned soil microbiome.

**Conclusion:**

Together, these findings revealed that beneficial microbiome feedbacks occur at low levels of plant-available iron, i.e. when plants grow in a suboptimal soil, but they are lost when plants are nutritionally well supported. These results underscore the importance of iron availability in soil for beneficial microbial feedbacks on plant growth and predict agronomic benefits of incorporating maize in crop rotations on low iron soils.

Video Abstract

**Supplementary Information:**

The online version contains supplementary material available at https://doi.org/10.1186/s40168-026-02493-8.

## Introduction

Plants condition their local soil environment by the release of root exudates [45]. This form of soil conditioning, modifying the physicochemical as well as microbial properties, contributes to plant-soil feedbacks (PSF) [2, 50], a phenomenon describing altered plant performance due to the previous generation of plants growing in the same soil. If an altered soil microbiome is the proximal cause for altered performance, then these plant responses are referred to as ‘microbiome feedbacks’ [26]. Such soil or microbiome feedbacks on plant growth, defence or development can have profound implications in practice (e.g., crop yields) or nature (e.g., ecosystem succession dynamics) [33]. In agriculture, such soil or microbiome feedbacks operate in crop rotations where the past crops impact the yield of the subsequent crop. Today, optimal rotation schemes take advantage of feedbacks that enhance soil fertility, promote beneficial microbial communities, suppress soil-borne pathogens and reduce plant pests [17, 29, 41]. Altering or co-cultivating crops started with Neolithic farming in the Near East [61] and more optimal rotation schemes continue to develop with continuous learning [38]. While farmers have learned and identified cropping sequences that exploit positive over negative feedbacks, the underlying mechanisms often remain unknown today.

Plant-soil and microbiome feedbacks are highly context-dependent. Feedbacks vary not only in strength but also in their direction (they can be negative or positive) depending on the soil environment. The high context-dependency is thought to result from the interplay between biotic (e.g., fauna, microbiota) and abiotic soil properties, including physical (e.g. structure, moisture) and chemical (e.g. nutrient availability, plant exudates) parameters [10]. Typically, feedback responses are not fully consistent *between* different fields [1], which is not surprising as different soil types differ in many ways such as in their physicochemical parameters. Feedback responses vary also in a more fine-grained manner due to local soil heterogeneity *within* fields. Farmers experience such context-dependent feedbacks when observing over- and under-yielding areas in their fields [32]. Deciphering the context-dependency, both within and between field soils, that affects strength and direction of plant-soil and microbiome feedbacks is of key importance towards understanding the underlying mechanisms.

Maize (*Zea mays* L.) and wheat (*Triticum aestivum* L.) are two of the most commonly grown crops worldwide [51]. Both crops are typically cultivated based on heavy use of agrochemicals, responsible for negative environmental impacts [36, 42], and therefore, in focus for more sustainable agricultural practices. Among these, maize-wheat rotations for instance become more widely practiced in Europe [36]. Like other sweet grasses, maize and wheat produce benzoxazinoids (BXs), a class of multifunctional plant secondary metabolites [39]. BXs are defence compounds against generalist insects, have antimicrobial activity, function allelopathically as phytotoxins, serve as phytosiderophores for iron uptake or alleviate abiotic stress by soil aluminium and arsenic [59]. Importantly, BXs are exuded from the roots to the surrounding soil, where they strongly alter the microbial communities [6, 11, 23, 30]. Maize secretes quantitatively more BXs than wheat and predominantly DIMBOA, DIMBOA-Glu and HDMBOA-Glu (see methods for full chemical names) [22, 23]. Thus, sweet grasses like maize and wheat condition the microbiomes in the surrounding soil by exudation of BXs.

BX-conditioned soil microbiomes (referred to as ‘BX_plus_ microbiomes’) drive differential feedbacks on the next plant generation as observed in experimental maize-maize [22, 23] and maize-wheat [5] rotations in the greenhouse. These conclusions are derived from comparing plant performance on BX_plus_ relative to the control ‘BX_minus_ soil’ (i.e., microbiome conditioned by BX-defective maize plants). For instance, wheat grew smaller but was more resistant to insects in response to the BX_plus_ microbiome [5]. Feedbacks driven by BX_plus_ microbiomes were also found for the model plant *Arabidopsis thaliana* (hereafter Arabidopsis) growing in maize conditioned soil in climate chambers [48]. The reference accession Columbia-0 grew larger, was developmentally more advanced and was more resistant to the fungal pathogen *Botrytis cinerea*. Furthermore, Arabidopsis revealed massive genetic variation in their growth feedbacks, allowing the identification of a plant immune receptor mediating these microbiome feedbacks on plant growth [25]. All above reported work on feedbacks of maize, wheat or Arabidopsis in response to BX_plus_ microbiomes was performed in controlled conditions using soil from the same field site (Changins, Switzerland). For the many experiments we repeatedly went back to this site and collected batches of soil from different spots of the field. Over the many experiments, we noticed strong variation in the strength of the feedbacks between soil batches and initially dismissed these observations as experiment-to-experiment variations [note: this study here now reveals that the variation in feedback strength is due to local soil heterogeneity from where the different soil batches were collected].

Complementary to the work in controlled conditions (paragraph above), we have tested the agronomic relevance of the feedbacks of maize BX_plus_ vs. BX_minus_ microbiomes on wheat in agronomically realistic conditions at two field sites [16, 17]. In the first season, we cultivated wild-type BX producing and BX-deficient maize lines to differentially condition the soil and then assessed in the following season the feedbacks of BX_plus_ vs. BX_minus_ microbiomes on winter wheat. In the first field site (Posieux), we found that soil conditioning by BXs increased wheat emergence, tillering, height and biomass and decreased insect herbivore abundance at the same time [17]. Importantly, these BX-induced microbiome feedbacks increased wheat yield by 4% without a reduction in grain quality in this maize-wheat rotation. These BX-induced microbiome feedbacks were confirmed in a repeat experiment at the second field site (Changins, same site as for soil collection)—but they were not apparent at first glance [16]. Averaged across the field, we did not detect field-scale feedbacks on wheat performance. However, taking the local soil condition into account, significant feedbacks were found along the physicochemical gradient existing in that field. For instance, wheat gradually responded to the BX_plus_ microbiome with varying heights along the soil gradient; i.e., BX_plus_ microbiome caused increased height at one and decreased height at the opposing end of the field gradient. Taken together, BX conditioned soil microbiomes drive feedbacks on wheat in agronomically realistic conditions, but they vary in strength and direction depending on local soil physicochemical heterogeneity.

**Fig. 1:**
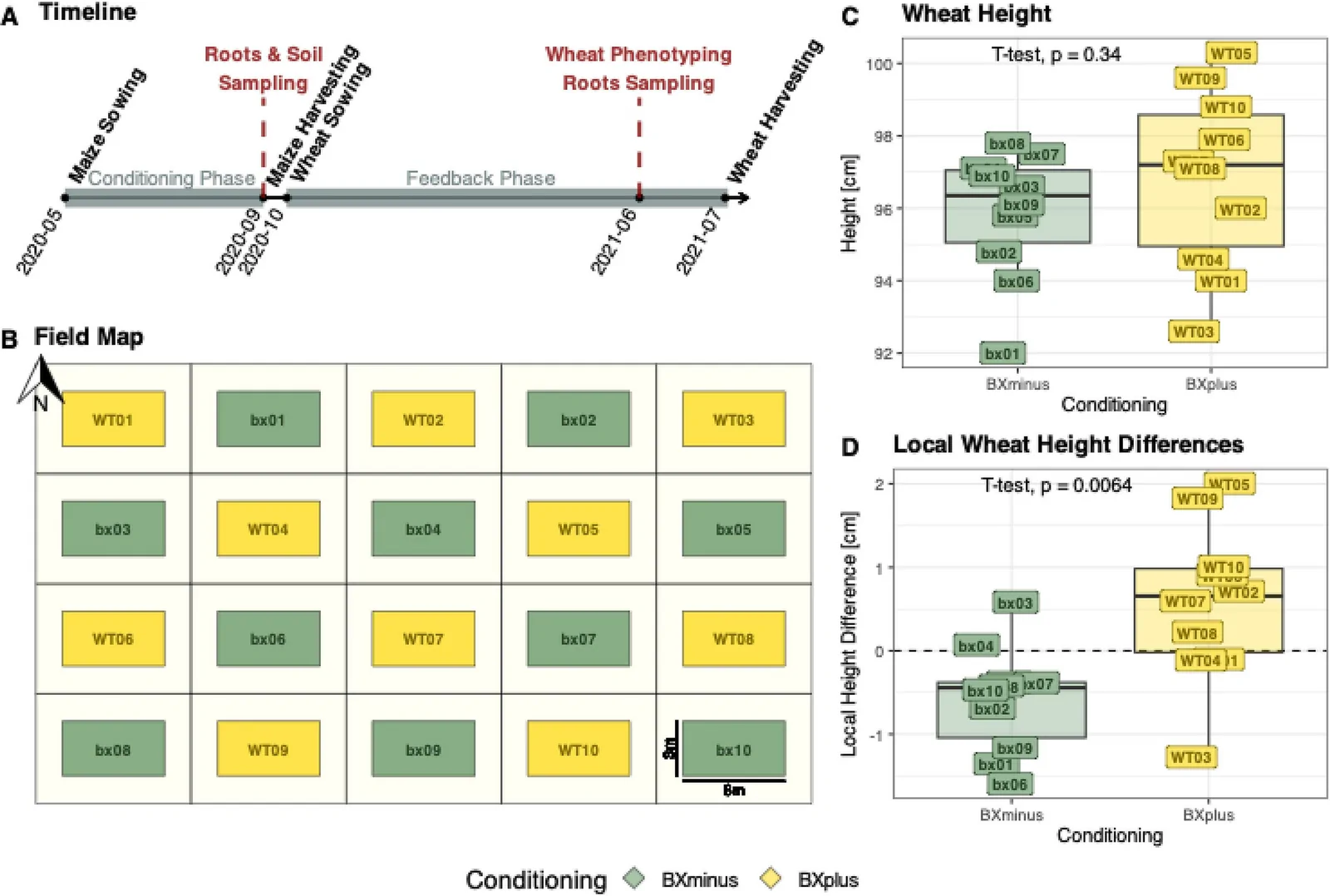
Maize-wheat plant-soil feedback experiment in Reckenholz. **A** At the Reckenholz field site a maize-wheat feedback experiment was performed. **B** A total of 20 3 × 6 m plots were established, half of it were conditioned with wild-type maize (BX_plus_ conditioning) and the other half with the benzoxazinoid-defective *bx1* mutant (BX_minus_ conditioning). Each plot was surrounded by a 3 m-wide buffer zone planted with *Phacelia*. **A** At the end of the conditioning phase, the maize root microbiome was sampled, physicochemical soil properties were measured and maize was harvested. Following the conditioning phase, wheat was grown on the conditioned soils. Wheat phenotypes were measured and wheat root microbiome was sampled towards the end of the feedback phase. The detailed phenotyping of wheat is described in Fig. S5 while here, plant height (**C**) is shown as an example. Wheat did not differ in height whether grown on BX_plus_ or BX_minus_-conditioned soils (t-test, *p*-value displayed in panel). **D** However, taking spatial field heterogeneity into account, i.e. calculating the “local height differences” by normalising each plot to its neighboring plots (see Methods and Fig. S2), wheat differs in height between BX_plus_ and BX_minus_ conditioned soils (t-test, *p*-value displayed in panel)

Here in this study, we tested the reproducibility of the aforementioned findings that soil conditioning by BX-producing plants increased wheat growth [17] and that these microbial feedbacks vary in strength and direction depending on soil heterogeneity [16]. Analogous to our two previous field studies, we conducted a third experiment at a new field site growing wild-type and BX-deficient maize to differentially condition the soil microbiomes and using wheat to score feedbacks (Fig. 1AB). We captured local soil heterogeneity in each replicated plot by measuring the physicochemical parameters and the microbial communities, which maize plants leave as legacy for wheat growth. Through multivariate, correlation and modelling analyses and combining the data from the three field experiments, we confirmed the differing microbial communities due to BX conditioning and that the strength of their feedbacks on wheat largely depended on the availability of soil iron levels. We extended our analysis to experiments with Arabidopsis where the strength of the feedbacks varied by soil batches. Like wheat, Arabidopsis showed an inverse relationship between soil iron and microbial feedbacks, which we could experimentally validate with iron supplementation. Overall, our work revealed that low levels of soil iron can be sufficient so that the BX-conditioned soil microbiomes promote growth as feedbacks on the subsequent plant generation.

## Material and methods

### Maize-wheat rotation experiment in the field

The setups of the field experiments in Changins and Posieux have been described earlier in Gfeller et al. [16, 17]. Here we document the specifications, plant material, setup and sampling of the new field experiment performed at Reckenholz (Zürich, Switzerland). The field is called ‘Schlag 109' (47° 25′ 45″ N, 8° 31′ 23″ E) and belongs to the research station Agroscope. This site has a loamy soil (clay ~ 21%, silt ~ 31% and sand ~ 46%) with pH 6.8 and an organic matter content of 3%. The more detailed physicochemical soil analysis is documented on GitHub (dataset in section B01). The field’s history was 3 years of fodder meadow (mixture of red clover and Italian ryegrass), and it was managed according to Swiss conventional agricultural practices by the field team of Agroscope.

The experiment consisted of a maize-wheat rotation with maize cultivated during the conditioning phase in 2020 and wheat in 2021 in the feedback phase (Fig. 1A). For soil conditioning, we grew wild-type maize (*Zea mays*; inbred line W22) and the benzoxazinoid-deficient mutant *bx1* in W22 background [49]. After clearing the preceding meadow with Roundup (8 L/ha) in autumn 2019, the seed bed was prepared in spring 2020 with a harrow, weeds controlled with herbicides (Aspect 1.5 L/ha, Banvel 4S 0.5 L/ha and Laudis 2 L/ha) and the seeds then directly sown with a seed drill (seeding: 28.05.2020). In total, we used 2′500 seeds wild-type and 2′750 seeds the *bx1* mutant maize to correct for the lower germination rate of the latter (determined ahead). Maize was sown in rows with 75 cm distance between rows. Wild-type and *bx1* mutant maize were alternately seeded with 10 replicate plots each following a chessboard design (Fig. 1B). Each plot measured 6 × 3 m ‘containing 4 rows of maize (Fig. S1A) and was surrounded by a 3 m buffer zone, where we planted *Phacelia*. The field was minerally fertilised with a total of 115 kg/ha N in form of urea applied in two doses. Harvest was after 14 weeks (harvest: 08.09.2020): in the centre of each plot, a maize plant was randomly selected, dried in the oven at 60° C for 48 h and the shoot dry weight was measured (Fig. S4A, number of replicates in Table S1). Similar to Gfeller et al. [16], we calculated local differences of the maize phenotypes by subtracting from the values of each BX_plus_ plot the mean values from their surrounding BX_minus_ plots and vice versa. With a t-test, we investigated for biomass differences between conditionings (Fig. S4B, more detailed explanation in Fig. S2, GitHub, section B02). At two locations per plot, we selected single maize plants of which we sampled the underneath soil (cores of 20 × 20 × 20 cm) and root systems (Fig. S1A). Both the soil and root samples were mixed per plot for subsequent analyses of physicochemical soil parameters and root microbiota profiles, respectively, as described below.

For the feedback phase (Fig. 1A, Fig. S1B), we then cultivated the winter wheat variety ‘CH Claro’ (referred as Claro; UFA Samen, www.ufasamen.ch; same variety as in Changins and Posieux). Seed bed preparation included a ploughing and harrowing of the field followed by direct sowing with a seed drill (seeding: 22.10.2020, 1.6 dt/ha). The wheat was sown in rows with 15 cm distance between the rows across all plots. The field was minerally fertilised with three applications of ammonium nitrate (1: 48 kg/ha of 24% N, 5% Mg, 6% S; 2: 60.5 kg/ha 27.5% N and 3: 24 kg/ha 24% N, 5% Mg, 6% S) equivalent to a total of 132.5 kg/ha N and the following plant protection products were applied: herbicides Globus 0.07 L/ha and Othello 1 L/ha and the insecticide Audienz 0.05 L/ha. On 11.06.2021, when all plants had reached the stage of fruit developing (BBCH 70–79), we selected in 3 lines 10 random wheat plants within a 4 m segment of the plots to get the mean plant height (cm), mean chlorophyll content (determined with a Minolta SPAD metre) and mean insect infestation by counting the number of larvae from the *Oulema melanopus* beetle on the two newest tillers. The aboveground biomass of 1 m of a wheat row was sampled, dried in the oven at 60° C for 48 h and the dry weight was determined (g/m) (Fig. S1B, Fig. 1C, Fig. S5ABC, number of replicates in Table S1). At two locations per plot, we selected single wheat plants from which we sampled the underneath root systems to profile bacterial communities as described below (Fig. S1B). After this, we re-established the plot outlines by mowing to keep a 4 × 1.5 m core-zone based on GPS coordinates. We finally harvested on 30.07.2021 the core zone and determined the kernel yield (g/m^2^) (Figs. S1B, S5D, number of replicates in Table S1, dataset on GitHub, section B05).

### Soil physicochemical analysis

The collection of soil cores was described above. Per plot 500 g of soil was sent to LBU Laboratories (Eric Schweizer AG, Thun; Switzerland) for analysis of physicochemical soil parameters. The same parameters as in Gfeller et al. [16, 17] were measured. Five plots could not be analysed due to a lack of soil (BX02, BX05, WT02, WT03, WT05). Despite the lack of these plots, both sample groups remain largely balanced with 7 BX_plus_ and 8 BX_minus_ plots. The reference methods used included a 1:10 (w/v) water (H₂O) extraction estimating plant-available (PA) nutrients, a 1:2.5 (w/v) extraction using water saturated with carbon dioxide (CO₂) measuring partly plant-available nutrients (PPA) and a 1:10 (w/v) ethylenediaminetetraacetic acid (EDTA) extraction that determines nutrients present as the plant-unavailable (UA) fraction in soil (dataset on GitHub, section B01).

### Maize and wheat root microbiome analysis

The collection of soil cores was described above. To prepare root microbiota samples, we followed Gfeller et al. [17] with the following changes: maize samples were stored at room temperature and immediately processed and wheat samples were stored at −20 °C. Wheat samples were lyophilised for 72 h. Root fragments were ground in 25 mL grinding jars with a 1.5 cm steel ball using a ball mill (Retsch, Germany) for 120 s by 30 Hz. One hundred twenty-five milligrams mg of root powder was used with the KF MagAttract Power Soil Kit (Qiagen, Germany) on a KingFisher robot (Thermo Fisher, USA) Samples were extracted in the double amount of extraction buffer for 10 min. DNA concentrations were measured with the AccuClear Kit (Biotium, USA). The DNA was diluted to a final amount of 1 ng template per PCR reaction.

We used the same microbiota profiling approach (described in Gfeller et al. [17]) as for our other field experiments in Changins and Posieux. In brief, this is a 2-step approach using the PCR primer 799‐F [9] and 1193‐R [3] and ITS1‐F [15] and ITS2‐R [55] for bacteria and fungi, respectively. PCR1 is performed with non-barcoded primers (bacteria: 25 cycles, fungi: 23) and after purification, PCR2 tags the PCR products with barcodes (same cycling conditions (see Gfeller et al. [17]) but with fewer cycles: bacteria 10, fungi 7). After library preparation (equimolar pooling, ligation of sequencing adapters, purification), samples were sequenced using a paired-end 300 bp v3 chemistry on an Illumina MiSeq instrument at the NGS platform of the University of Bern (ngs.unibe.ch). The raw sequencing data is made available at ENA (see below).

### Maize-wheat feedback experiments

All analyses of the maize-wheat experiments were conducted for the field in Reckenholz alone and also combined with the data from the previous field experiments in Changins [16] and Posieux [17]. Of note, expanding the new data from Reckenholz with the two existing datasets enabled new integrative analyses beyond site-specific variation in feedbacks and towards predicting PSFs more generally in the context of soil chemistry and microbiomes.

#### Preprocess of maize and wheat root microbiome data

Root microbiome raw sequencing (available on ENA, see below) data from Changins, Posieux and Reckenholz were combined. Then we followed the same pipeline as described in Gfeller et al. [17] to infer exact amplicon sequencing variants (ASVs) and assign taxonomies. These calculations were performed at sciCORE (http://scicore.unibas.ch/), the scientific computing centre of the University of Basel (metafile and code are on GitHub A, the processed sequencing dataset in section B03, B09). We used the R package vegan (version 2.6.8) [40], followed the recommendation of Weiss et al. [54] and normalized the samples by rarefying at thresholds of 9.000 and 1.000 bacterial and fungal sequences, respectively (Fig. S3). Samples with less sequences and samples detected as outliers by the CLOUD method (nearest neighbours = 40%, outlier percentile = 0.1) [37] were discarded (Table S1). The 1′000 reads for fungi may appear low but was chosen because the Changins dataset is from earlier sequencing. Although fungal diversity is not fully captured in all samples (dominant taxa are captured, but not the rare ones), the downstream modelling should not be much affected due to stringent filtering (see below).

#### Maize root microbiome analysis

**Fig. 2:**
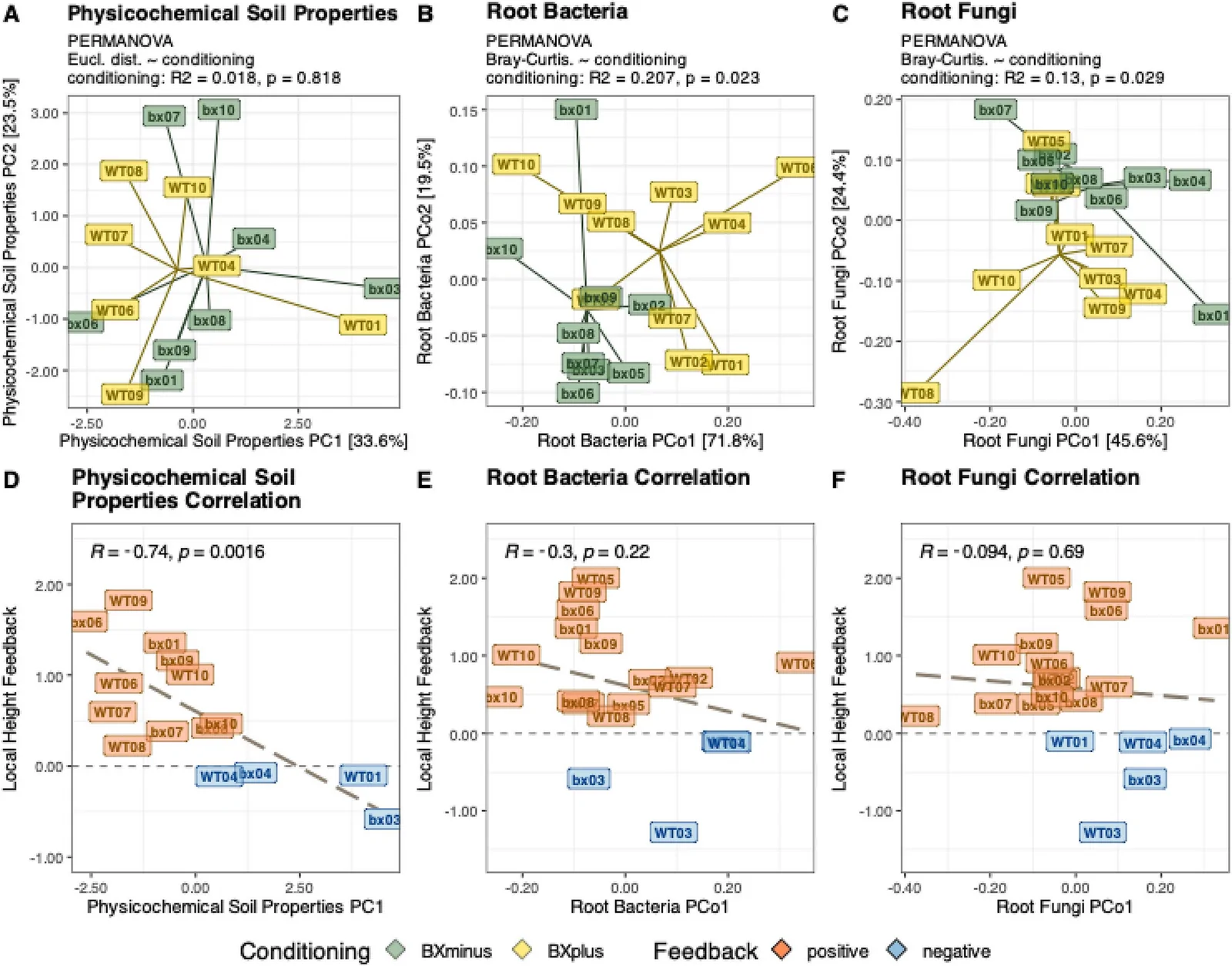
Soil properties, maize microbial communities and their relationships to wheat’s local height feedbacks in Reckenholz. **A** Principal component (PC) Analysis visualises the variation in physicochemical soil properties (Euclidean distance, PC axes 1 and 2 with % of variation explained are shown) while Principal Coordinates (PCo) Analysis depicts variation among **B** bacterial and **C** fungal community profiles of maize roots at the end of the conditioning phase (Bray–Curtis distance, PCo axes 1 and 2 with % of variation explained are shown). The effects of conditioning were tested for soil physicochemical parameters and bacterial and fungal ASVs using permutational analysis of variance (PERMANOVA), with the model and corresponding R.^2^- and *p*-values displayed above the plots. Next, we tested the interdependence of wheat feedbacks with the soil chemical or microbial properties. The axes with highest correlation coefficient, explaining at least 10% of variation, were chosen for display (see Table S2). The local height feedbacks (all local differences expressed relative to BX_minus_) correlated best with **D** the first PC axis of the physicochemical soil parameters, **E** the first PCo axis of bacterial communities, and **F** the first PCo of fungal communities. The corresponding Pearson’s correlation coefficients (R) and *p*-values are indicated at the top of each panel. Colors represent the conditioning treatments (**A**-**C**) and feedback directions (**D**-**F**)

**Fig. 3:**
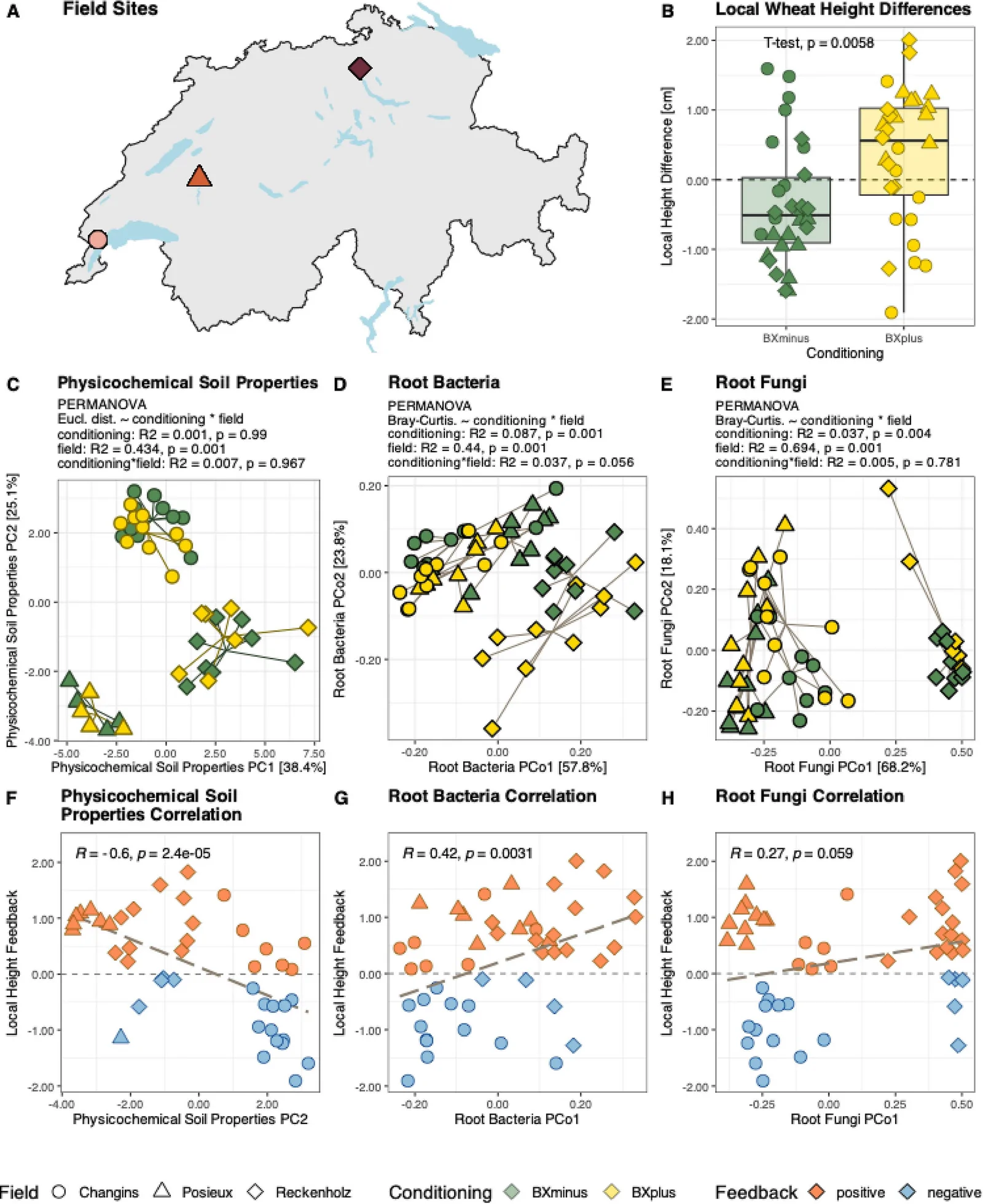
Combinatory analysis of soil properties, maize microbial communities and their relationships to wheat’s local height feedbacks across three field experiments. **A** We have conduced plant-soil feedback experiments with wild-type and benzoxazinoid‐deficient maize followed by wheat at the three field sites Changins, Posieux and Reckenholz in Switzerland. **B** We found increased local height differences for wheat grown on soil conditioned with WT-maize compared to wheat grown on *bx1* conditioned soil (t-test, *p*-value displayed in panel). **C** Principal component (PC) Analysis was used to display the variation in physicochemical soil properties, as measured at the end of the conditioning phase (Euclidean distance, PC axes 1 and 2 with % of variation explained are shown). Principal Coordinates (PCo) analysis was used to visualise the variation among **D** bacterial and **E** fungal community profiles of maize roots at the end of the conditioning phase (Bray–Curtis distance, PCo axes 1 and 2 with % of variation explained are shown). The differences between sites, the effects of soil conditioning and their interaction were tested using permutational analysis of variance (PERMANOVA), with the model, R^2^- and *p*-values reported above the plots. Next, we tested the interdependence of wheat feedbacks with the soil chemical or microbial properties using correlation analysis. The axes with highest correlation coefficient, explaining at least 10% of variation, were chosen for display (see Table S3). The local height feedbacks (all local differences expressed relative to BX_minus_) correlated best with **F** the second PC axis of the physicochemical soil parameters and the first PCo axes of the **G** bacterial and **H** fungal communities. The corresponding Pearson’s correlation coefficients (R) and *p*-values are indicated at the top of each panel. Colors represent the conditioning treatments (**B**-**E**) or feedback directions (**F**–**H**) and shapes represent field site

Since microbial community data are compositional, sparse and characterised by many zeros, community effects on beta diversity were tested with permutational analysis of variance (PERMANOVA, 999 permutations) on Bray–Curtis distances. For Reckenholz plots, we tested for differences in conditioning and visualised it by the first two axes of a principal coordinates analysis (PCoA, Fig. 2BC, number of replicates in Table S1). Taking all field sites together, we investigated for differences in conditioning, field and conditioning-field interaction and showed the results again by plotting the first two axes of a PCoA (Fig. 3DE, number of replicates in Table S1). Dataset, code and analysis documentation are provided on GitHub, section B03.

#### Physicochemical soil parameters

We investigated for differences in conditioning, soil and their interaction. Since these measurements are continuous and have comparable scales, we used a PERMANOVA (999 permutations) on Euclidean distances and displayed results in a Principal Component Analysis (PCA, Figs. 2A, 3C, number of replicates in Table S1), and as biplot (Fig. S7, number of replicates in Table S1) with the package FactoMineR (version 2.11) [31], and the package factoextra (version 1.0.7) [28]. Dataset, code and analysis documentation are provided on GitHub, section B01.

#### Benzoxazinoid analysis

We measured the concentration of 2,4-dihydroxy-7-methoxy-1,4- benzoxazin-3-one (DIMBOA), 2-O-β-D-glucopyranosyl-2,4-dihydroxy-7-methoxy-(2H)−1,4-benzoxazin-3(4H)-one (DIMBOA-Glucose) and 2-hydroxy-4,7-dimethoxybenzoxazin-3-one (HDMBOA-Glucose) from Changins and Posieux sites at the end of the conditioning phase. Methods were described in Gfeller et al. [16, 17]. Although BX concentrations were quantified for all plots, only data from BX_plus_ conditioned plots were used in the present analyses, as BX levels in BX_minus_ plots were previously shown to be negligible at these sites (Fig. S12) [16, 17]. No benzoxazinoids were measured in Reckenholz. For details see GitHub, section B04.

#### Plant feedbacks

The raw measures of the wheat phenotypes (height, chlorophyll, biomass, insect infestation and kernel yield) were normalised by z-score normalisation per field. Following Gfeller et al. [16], we calculated the local differences of the wheat phenotypes to take the local soil condition into account. For this, the wheat phenotypes of a plot are expressed relative to the mean of the adjacent plots, i.e. subtracting from the wheat phenotypes of each BX_plus_ plot the mean from the surrounding BX_minus_ plots and vice versa (see graphic explanation in Fig. S2). The Fig. S2 reports the number of neighbouring plots that contribute to each value and discloses the neighbourhood structure of the data. While the calculations of local differences reduce soil heterogeneity (e.g., by shared spatial gradients), they introduce some level of data dependence. Hence, the metric of local differences shall be interpreted in the context of a spatially structured field design rather than fully independent observations. Since the local differences still fulfilled the assumptions of normality, we examined them like the raw phenotype measure with t-tests comparing the two conditionings (BX_plus_ vs. BX_minus_, Fig. 1D, Fig. S5E-H, Fig. 3B, Fig. S6F-J, number of replicates in Table S1, GitHub section B05).

Of note, the local difference values have “different directions” as the data of a plot is expressed relative to its neighbouring plots (i.e., local differences are either expressed relative to BX_plus_ or relative to BX_minus_). To define feedbacks with the “same direction”, we inversed the local differences of wheat phenotypes in the BX_minus_ plots (i.e., multiplied by −1). Like this all local differences become expressed relative to BX_minus_ and we refer to them as local feedbacks. The local feedback of a phenotype has a positive value (indicated in the figures with orange color) when wheat grew better on BX_plus_ compared to BX_minus_ conditioned plots and a negative value (figures: blue color) when wheat grew less on BX_plus_ compared to BX_minus_ plots. This transformation to unidirectional feedbacks is essential for the subsequent correlation analyses.

We correlated local wheat phenotypes feedbacks with the PCA axes from the physicochemical soil analysis, by performing a Pearson correlation test. The local feedback data was excluded from the five plots without physicochemical soil measurements. For the Posieux data—where soil was pooled for physicochemical soil properties—we calculated the mean local feedbacks according to the pooling of soil samples (Fig. 2D, Table S2, Fig. 3F, Table S3, number of replicates in Table S1, GitHub section B06). Local wheat phenotype feedbacks were also correlated to the PCoA axes of bacteria and fungi. Samples without bacterial or fungal root microbiome measurements and samples which were discarded during microbial analysis were removed (Fig. 2EF, Table S2, Fig. 3GH, Table S3, number of replicates in Table S1, GitHub section B07).

#### Modelling

For the aim to identify the parameter(s) explaining strength and direction of wheat feedbacks, we modelled the physicochemical soil measures and the abundant ASVs of the maize microbial communities relative to the local height feedbacks across the three field sites. Samples were removed because paired data of physicochemical soil parameters, bacterial abundance or fungal abundance was not available. Table S1 summarizes the final replicate numbers used for modelling from each site and data type. For samples from the Posieux field, we used as described above the mean local height feedbacks. According to this, we also calculated and used mean microbial abundances. We tested for collinearity in the physicochemical soil parameters by calculating a correlation matrix and variance inflation factors (Fig. S8). Due to the high dimensionality of our dataset (more explanatory variables than samples) and the presence of substantial multicollinearity among variables, we used least absolute shrinkage and selection operator (LASSO) regression (package glmnet, version 4.1.8) [13] to select the best prediction model [14]. As explanatory variables, we included all measured physicochemical soil parameters and all root bacterial and fungal ASVs that both, occurred in at least 4 samples and reached a minimal relative abundance of 0.15% in the dataset. This stringent filtering helps to focus on abundant and reproducible taxa and at the same time reduces impacts of lower sequencing depth as in the case of the Changins data set (Fig. S3). Furthermore, a stringent threshold helps to balance biological resolution with statistical and computational constraints of the LASSO framework. The model would be heavily overfitted without filtering and only reducing the number of taxa (6,203 to 132 bacterial ASVs and 776 to 56 fungal ASVs) substantially improved model performance and interpretability. Using k-fold cross-validation, we determined the optimal value of the regularisation parameter λ (L1 penalty). We selected λ such that the model error remained within one standard error of the minimum cross-validation error. Variables with non-zero coefficients were retained as predictors.

**Fig. 4:**
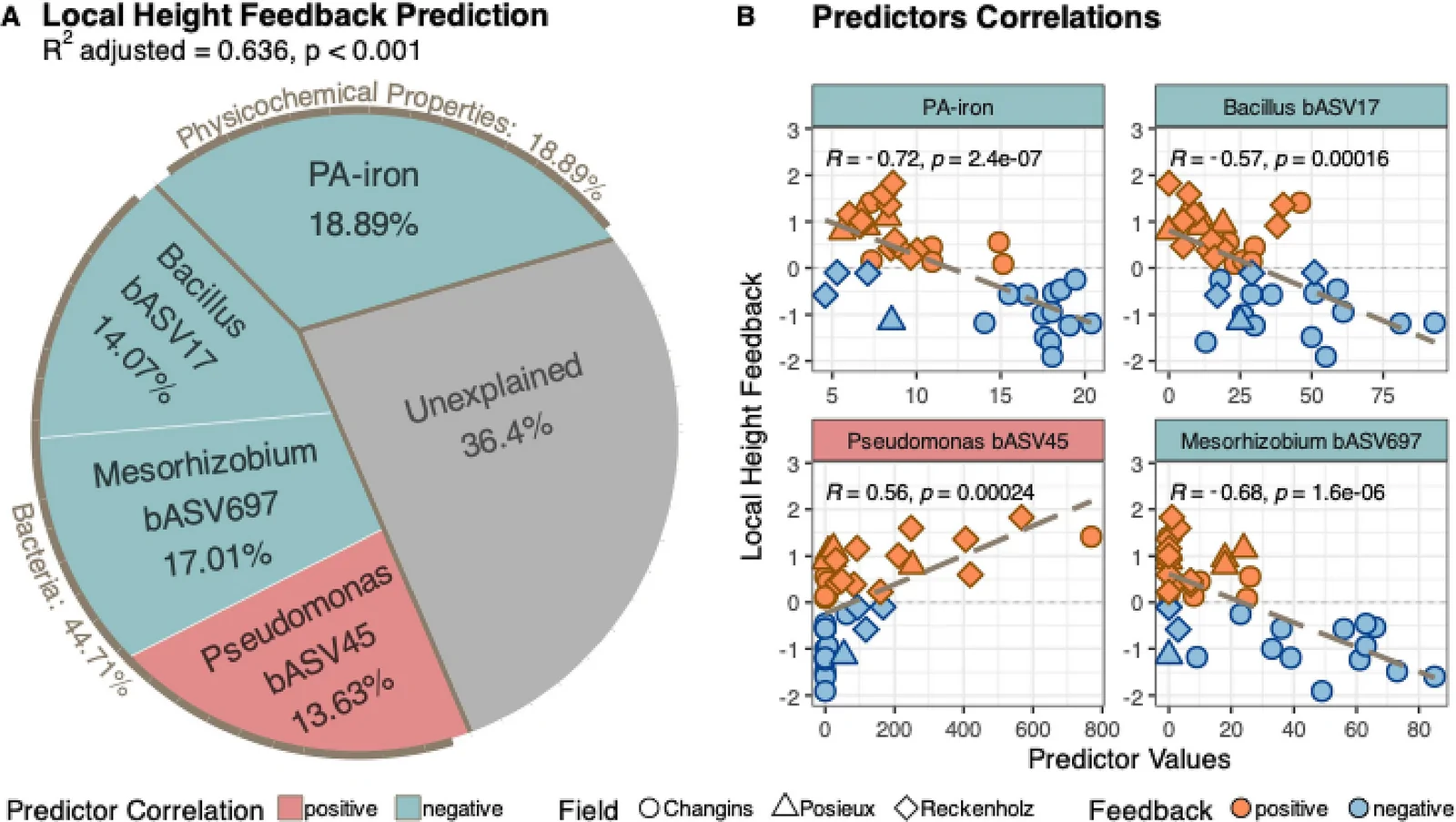
Modelling local height feedback using physicochemical soil properties, bacterial ASVs and fungal ASVs. **A** Predictor selection for explaining local height feedback was done by a least absolute shrinkage and selection operator (LASSO) regression with a repeated subsample-based variable stability. The total model performance was adjusted by the number of selected predictors. Adjusted R^2^ and *p*-value are indicated above the plot. The Voronoi plot shows selected predictors. The proportion of explained variation based on adjusted R^2^ in local height feedback is labelled for each predictor and for predictor groups. **B** Local height feedback (all local differences expressed relative to BX_minus_) was correlated with each selected predictor. Pearson's correlation coefficient (R) and *p*-value are labelled at the top of each panel. Colours represent predictor correlation directions (**A**) and feedback directions (**B**). Shapes represent field sites

Given that model selection can be unstable in the presence of high multicollinearity and sparsity among predictors, as numerous zero values, we did a repeated-subsampling stability analysis to assess the robustness of variable selection and mitigate seed dependency [19]. Inspired by the approach of Meinshausen and Bühlmann [35], we ran the model 500 times using 80% of the samples in each iteration, without replacement. While re-estimating λ in each iteration prevents formal control of the expected number of false positives, we report the frequency of variable selection across iterations as an indicator of stability. Predictors selected in less than 25% of iterations were considered unstable and were excluded (Fig S9A). The remaining predictors were categorised based on selection stability as low (25–50%), moderate (50–75%) or high (75–100%) (Fig S9B). Pearson correlation tests were used to determine the correlation for each retained predictor with the local height feedbacks. Then, we evaluated the adjusted R^2^ and *p*-value of the final model using analysis of variance (ANOVA), and calculated adjusted relative importance values for each predictor by averaging over predictor orderings (Fig. 4). Finally, we complemented the LASSO analysis with a mixed-effects model, including field site as a random effect in the null model. We then added the predictors, either individually or all together, and compared the AIC of each tested model with that of the null model. Model improvement was assessed using likelihood ratio (χ2) tests (Fig. S10). (package relaimpo, version 2.2.7) [18]; see GitHub, section B08).

#### Wheat root microbiome analysis

The LASSO modelling approach described above identified bacterial predictors from the maize root microbiome whose abundances were significantly correlated with local height feedbacks. Pearson correlation tests were then used to determine whether the abundances of the same ASVs in the wheat root microbiome were also correlated with local height feedbacks (Fig. S11). Dataset, code and analysis documentation are provided on GitHub, section B09.

### Maize-Arabidopsis feedback experiments

Multiple independent maize – Arabidopsis feedback experiments were performed. We combined their data to investigate them collectively. Details on GitHub, section B010.

#### Experimental setups

For each experiment, field soil was conditioned by growing wild-type maize and *bx1* mutants for 12 weeks. Conditioning was either carried out directly in the field or soil was previously collected, sieved (1 cm mesh) and maize was grown in individual pots in the greenhouse or Phytotron chambers. For details see Table S4, Stengele et al. [48] and Janse van Rensburg et al. [25]. At the end of the conditioning phase, conditioned BX_minus_ and BX_plus_ soils from repeat field plots or pots were pooled by maize genotype and experiment. A 500 g sample of each conditioned soil from each soil batch was sent to LBU Laboratories (Eric Schweizer AG, Thun; Switzerland) for analysis of physicochemical soil parameters. The same parameters as for the previously described Reckenholz samples were analysed. After the conditioning phase, the soil was mixed with 20% autoclaved quartz sand and pots (5.5 × 5.5 × 5 cm) were filled by weight. The pots were sown with stratified seeds of the reference accession Columbia-0 (3 days at 4 °C). Plants were grown in Percival growth chambers (CLF Plant Climatics, Wertingen, Germany) with 10 h of light (100 μmol m^−2^ s^−1^, 21 °C, 60% relative humidity) and 14 h of darkness (0 μmol m^−2^ s^−1^, 21 °C 18 °C, 60% relative humidity). They were watered 3 times a week by weight and randomized once per week. Plants were either not fertilised, fertilised with 10 mL of 1:3 times diluted ½ strength Hoagland solution at 21 days or 14 days and 21 days after germination or fertilized with 5 ml of undiluted ½ strength Hoagland solution 21 d and 35 d after germination. Plants were photographed 40 ± 2 days after germination. Detailed information for each experiment is given in Table S4.

#### Feedback analyses

**Fig. 5:**
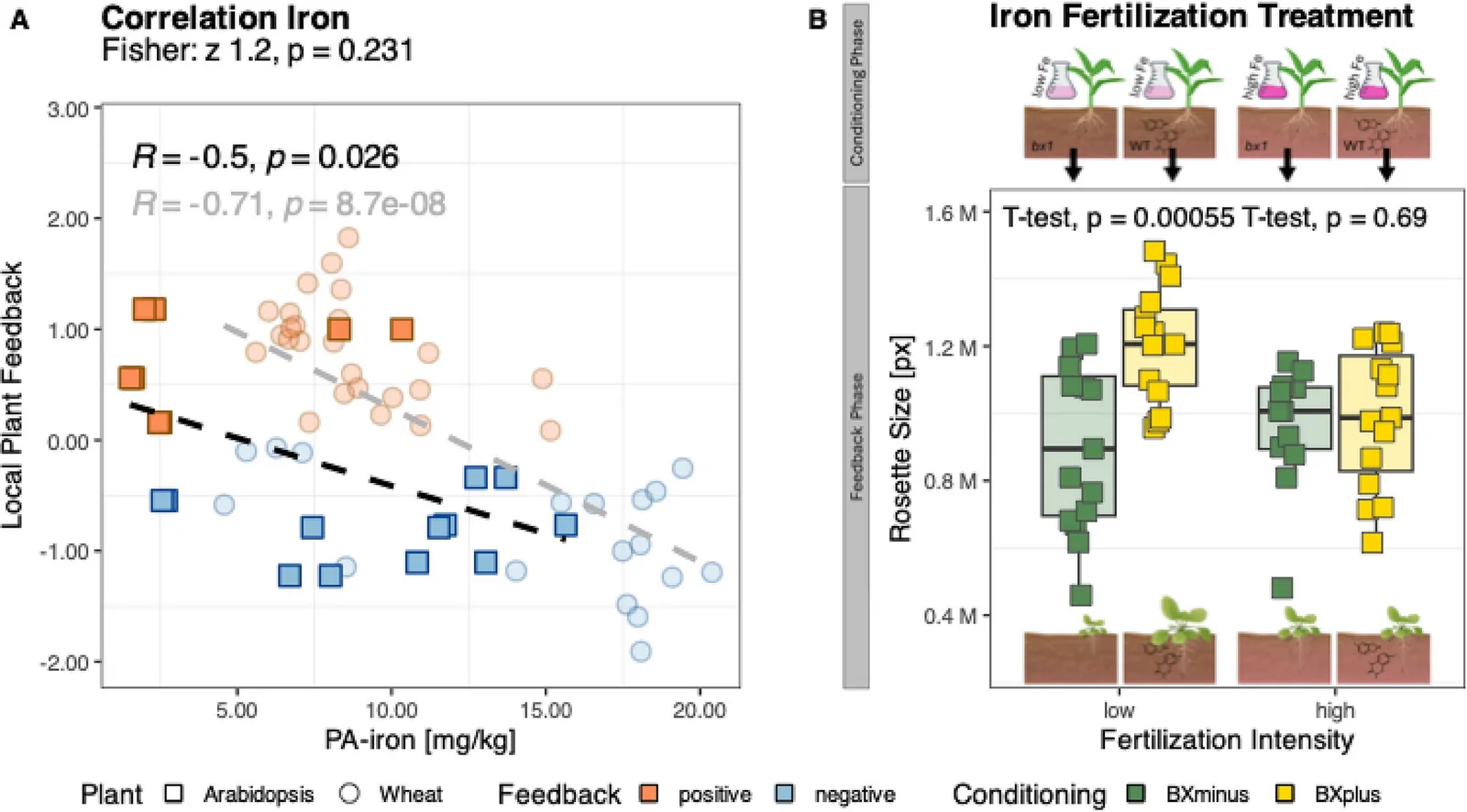
Meta-Analysis of maize- Arabidopsis plant-soil feedback experiments. **A** Local rosette feedback of Arabidopsis, respectively local height feedback from wheat, was calculated and correlated with plant-available iron (PA-iron). Pearson's correlation coefficient (R) and *p*-value are labelled at the top. With a Fisher test it was investigated for differences in the correlation coefficients between the two independent groups of Arabidopsis and wheat. Test statistic z and *p*-value are labelled above the plot. **B** At the end of the feedback phase, differences in rosette size of Arabidopsis grown on BX_plus_ and BX_minus_ conditioned soil were tested using a t-test. Colours represent feedback directions (**A**) and conditioning treatments (**B**). Opacities and shapes refer to maize-wheat experiments and maize-Arabidopsis experiments

To determine Arabidopsis growth, plant-photographs were processed using the ARADEEPOPSIS 1.3.1 software [24] to compute the rosette area for each sample. Rosette areas were normalised using z-score normalisation within each experiment. Similar to local height differences used in the wheat experiments, we calculated rosette differences for Arabidopsis. For each data point x within an experiment e, the rosette differences was calculated as ${rosette\_differences}_{Xe}=x_e-mean\left(y_e\right)$, where ($y_{\mathrm e}$) represents all other data points in the same experiment but of the other conditioning. Then we calculated the rosette feedback by multiplying BX_minus_ conditioned rosette differences by −1 (Fig. S13). Rosette feedbacks were correlated to PA-iron by performing a Pearson correlation test. To investigate for differences in the correlation coefficients between PA-iron local rosette feedback correlation and PA-iron local height feedback correlation, a Fisher test for independent groups was performed (Fig. 5A, package cocor, version 1.1.4) [12].

#### Iron supplementation experiment

Finally, we performed an iron supplementation experiment using the low-iron soil batch BS09. For the low iron treatment, we supplement soil during the conditioning phase with 100 mL of 0.2% Plantaaktiv Typ K (Hauert HBG Duenger AG, Grossaffoltern, Switzerland) and 0.001% Sequestrene Rapid (Maag, Westland Schweiz GmbH, Dielsdorf, Switzerland). For the high iron treatment, the first 4 weeks of conditioning was done identical to the low iron treatment before increasing the concentrations to 200 mL of 0.2% Plantaaktiv Typ K and 0.02% Sequestrene Rapid for the remaining 8 weeks of the conditioning phase. For both fertilisation treatments, a t-test was used to test for differences in rosette size of Arabidopsis grown on BX_plus_ and BX_minus_ conditioned soil (Fig. 5B).

### Hardware and software

If not explicitly mentioned, analyses were performed in R 4.5.1 [43]. For data organisation and transformation, the tidyverse-package collection 2.0.0 [56], gtools 3.9.5 [53] and phyloseq 1.54.2 [34] were used. Figures were created with ggplot2 3.5.2 from the tidyverse and the data-visualization packages cowplot 1.1.3 [58], ggpubr 0.6.0 [27], gghx4 0.3.0 [4], ggnewscale 0.5.1 [8], scales 1.4.0 [57], terra 1.8.54 [21], tidyterra 0.7.2 [20] and geomtextpath 0.1.5 [7]. Tables were visualized by the package kableExtra 1.4.0 [60].

### Availability of data and materials

The raw microbiome sequencing data is deposited at the European Nucleotide Archive (http://www.ebi.ac.uk/ena) under the study accessions PRJEB59165, PRJEB53704 and PRJEB94164. Datasets and code used in this study to analyse microbiome sequencing data, to do statistical analyses and to create figures are available on GitHub (https://github.com/PMI-Basel/Waelchli_et_al_Reckenholz_field_experiment). The GitHub repository has two parts with “A” containing information and bash and R code for microbiome analysis as follows: (A1) Metafile containing barcode, primer and sample assignment information (A2) raw sequencing file names and (A3) code to analyse microbiome sequencing data. The part “B” contains the raw data and R code for the following analyses: (B01) soil chemistry, (B02) maize conditioning, (B03) statistic analysis of maize root microbiome, (B04) BX soil concentrations, (B05) wheat feedback, (B06) local wheat feedback – soil chemistry interaction, (B07) local wheat feedback – conditioning microbiome interaction, (B08) modelling approach, (B09) statistic analysis of wheat root microbiome, (B10) Arabidopsis feedback experiments.

## Results

### Wheat growth depends on local soil condition

To assess the role of soil conditions on wheat growth, we performed a third field trial (site: Reckenholz), where analogous to the two previous experiments (sites Posieux and Changins), we first cultivated wild-type maize (inbred line W22) and the benzoxazinoid‐deficient mutant *bx1* (in W22 background) to differentially condition the soil (Fig. 1AB). Consistent with Posieux, field performance of wild-type and the *bx1* mutant plants was similar (Gfeller et al. [17], Fig. S4). Then, we assessed the growth of the winter wheat variety Claro in response to BX_plus_ vs. BX_minus_ soil microbiomes in the following season. Similar to the heterogeneous field in Changins [16], no field-scale effects were observed at Reckenholz while wheat performance differed significantly when considering the local differences (data of a plot expressed relative to the mean of adjacent plots; takes local soil condition into account, see Methods). We exemplify this conclusion with wheat height (Fig. 1CD) and document the other wheat phenotypes in Fig. S5. Feedbacks on plant height revealed no difference between the two conditioned soils across the entire field (*p* > 0.05, Fig. 1C), whereas the differences in *local* height revealed wheat to grow better on BX_plus_ compared to BX_minus_ soils (*p* < 0.01, Fig. 1D). Effects on local differences were also seen for chlorophyll content and the agronomically important kernel yield, but not biomass and insect infestation (Fig. S5). Thus, we found also for the Reckenholz site that soil conditioning by BX-producing plants increased wheat performance in a local soil context-dependent manner.

### Local physicochemical soil heterogeneity correlates with strength of feedbacks

Finding that wheat performance was affected by local soil condition (Fig. 1, Fig. S5), the next step was to search the soil parameters that could explain the local differences in growth. For this, we analysed in each plot the physicochemical parameters and the bacterial and fungal communities that maize plants leave as legacy for wheat growth. We used permutational analysis of variance (PERMANOVA) for quantification of effect sizes (approximated based on R^2^ values) and unconstrained ordination for visualisation. The physicochemical parameters did not differ between soils conditioned by wild-type or *bx1* maize plants (R^2^ = 1.8%, *p* > 0.05, Fig. 2A). In contrast, larger effects sizes and significant differences between wild-type and *bx1* maize plants were found in root bacterial (R^2^ = 20.7%, *p* < 0.05, Fig. 2B) and fungal communities (R^2^ = 13.0%, *p* < 0.05, Fig. 2C). Thus, the differential conditioning affected the microbial but not the physicochemical parameters of the soil.

Next, we evaluated to which degree wheat growth depended on the soil physicochemical or microbial parameters in each plot. For this, we calculated for each wheat phenotype the local feedbacks (all local differences expressed relative to BX_minus_; defines unidirectional feedbacks, see Methods) and conducted correlation analyses using the ordination axes of the physicochemical parameters and the microbial communities explaining at least 10% of variation (Table S2). While no relationships were found for chlorophyll content, biomass, insect infestation and kernel yield, the wheat's local height feedbacks correlated strongly and significantly with the local soil parameters (physicochemical soil chemistry PC1; *R* = −0.74, *p* < 0.01, Fig. 2D) while this was not the case with the microbial communities (bacteria: PCo1, *R* = −0.30, *p* > 0.05, Fig. 2E; fungi: PCo1, *R* = −0.09, *p* > 0.05, Fig. 2F). Taken together, BX-producing and defective maize lines differentially condition the microbial communities but the local physicochemical soil parameters are strongly associated with the strength of the microbial feedbacks on wheat height.

### Dependence of wheat feedbacks on soil parameters is consistent across multiple fields

Next, we integrated the Reckenholz finding, that local soil heterogeneity links with varying feedback strength, to our earlier field experiments conducted at Posieux [17] and Changins [16] (Fig. 3A). Analogous to above but for all three field experiments combined, we analysed the local wheat height differences, physicochemical soil parameters, the microbial communities of maize plants and their interdependence. Wheat performance was significantly enhanced in BX_plus_ compared to BX_minus_ conditioned plots (*p* < 0.01, Fig. 3B). We noticed substantial variation in local height differences within each field soil (Fig. S6A, other phenotypic traits and local differences are reported in Fig. S6B-J) and examined also the soil parameters across the three fields.

The physicochemical soil parameters differed significantly between the three field sites (PERMANOVA: R^2^ = 43.4% of the variance between sites, *p* < 0.01; Fig. 3C). This is not surprising with three different soil types: Reckenholz being silt-rich, Changins clayey, and Posieux sandy (Fig. S7A). It is noteworthy that Changins soil contained more than twice as much plant-available (PA) iron compared to the other fields (Fig. S7B). Consistent with the findings above (Fig. 2A) and the earlier two field experiments [16, 17], soil conditioning by BX exudation did not impact the physicochemical parameters (PERMANOVA: R^2^ = 0.001%, *p* > 0.05; Fig. 3C).

Next, we compared the microbial communities that maize plants leave as a legacy for wheat growth across the three field sites. Again, not surprising with three different soil types, the microbial communities significantly differed between the sites (PERMANOVA: bacteria: R^2^ = 44.0%, *p* < 0.01, Fig. 3D, fungi: R^2^ = 69.4%, *p* < 0.01; Fig. 3E). In contrast to the physicochemical soil parameters, the microbial communities were impacted by BX exudation: BXs caused an average shift of 8.7% in bacterial (*p* < 0.01) and 3.7% in fungal (*p* < 0.01) root communities. These findings are also consistent with the separated analyses of the field experiments (Fig. 2BC, Gfeller et al. [16, 17]).

Finally, we examined the interdependence of the varying strength of local feedbacks (Fig. 3B) with the variations in the soil properties across the three field sites. For this, we correlated the local feedbacks with the ordination axes of the physicochemical or microbial communities (Table S3). The strength of local height feedbacks correlated most strongly with the physicochemical soil parameters (PC2, *R* = −0.6, *p* < 0.001; Fig. 3F) and to a lesser extent with the maize bacterial communities (PCo1, *R* = 0.42, *p* < 0.01, Fig. 3G) but not with fungal communities (PCo1, *R* = 0.27, *p* > 0.05, Fig. 3H). The combinatory analysis reinforced the conclusion of the Reckenholz site be valid across the three fields: BX-producing and defective maize lines differentially condition the microbial communities. But locally varying soil physicochemical parameters are strongly associated with variation in wheat’s feedback strength, whereas local bacterial communities show a weaker association and fungal communities show no detectable association.

### Plant-available iron is the strongest predictor of local height feedbacks

To identify the explanatory parameter(s), we modelled the physicochemical soil measures and the abundant ASVs of the maize microbial communities relative to the local feedbacks across the three field sites. Although the local feedbacks of all measured wheat phenotypes varied significantly with one or more soil parameters (Table S3), we focus in the following on the local height feedbacks because they had the strongest individual relationship (correlation coefficient *R* = −0.6). We used repeated subsample-based LASSO regression with stability analysis (step 1) followed by stability-based filtering (step 2, see methods; Fig. S9A). A total of 63.6% of the variation in wheat height feedbacks could be explained by four parameters. The most reliable predictor was PA-iron, explaining 18.9% of variation and having a strong stability of 91.4%. Three weakly stable bacterial predictors explained together around 45% of variation and belong to the genera *Bacillus* (bASV17, explained variation: 14.1%, stability: 33.4%), *Pseudomonas* (bASV45, v: 13.6%, s: 33%) and *Mesorhizobium* (bASV697, v: 17.0%, s: 46.8%). No fungal predictor was found (Fig. 4A, Fig. S9B). Complementary mixed-effects model analyses, including field site as a random effect, supported these findings. Adding each individual predictor improved model performance compared to the null model, with the best performance observed when all predictors were included simultaneously (Fig. S10).

PA-iron was negatively correlated with local height feedbacks (*R* = −0.72, *p* < 0.001; Fig. 4B)—i.e. the fewer iron there is available in soil, the more positive the local height feedback is on wheat. bASV17 and bASV697 were also negatively correlated with the local height feedback (bASV17: *R* = −0.57, *p* < 0.001; bASV697: *R* = −0.68, *p* < 0.001) while bASV45 showed a strong positive correlation (*R* = 0.56, *p* < 0.001). By analysing the subsequent wheat microbiome towards the end of the feedback phase, bASV17 and bASV697 are still negatively correlated with the local height feedbacks (bASV17: *R* = −0.46, *p* < 0.01; bASV697: *R* = −0.62, *p* < 0.001) while bASV45 is no longer correlated (*R* = 0.01, *p* > 0.05; Fig. S11). Interestingly, we also found that PA-iron concentrations, bASV17 and bASV697 abundances were strongly correlated with the soil BX concentrations in BX_plus_ conditioned soil samples, while this was not the case for bASV45 (Fig. S12; DIMBOA (PA-iron: *R* = 0.82, *p* < 0.001; bASV17: *R* = 0.73, *p* < 0.05, bASV697: *R*: = 0.74, *p* < 0.001; bASV45: *R* = −0.36, *p* > 0.05) and DIMBOA-Glucose (PA-iron: *R* = 0.82, *p* < 0.001; bASV17: *R* = 0.73, *p* < 0.05, bASV697: *R* = 0.71, *p* > 0.05; bASV45: *R* = −0.34, *p* > 0.05). Summarising, we identified one soil parameter, PA-iron, and three maize bacteria, of which PA-iron was the strongest and most reliable predictor to explain the variation in local height feedbacks of wheat.

### Low levels of PA-iron link with beneficial microbial feedbacks on plant growth

The finding that PA-iron in soil is strongly associated with the strength of wheat feedbacks was reminiscent of the field experiment in Changins, where the chemical gradient in soil was particularly driven by high levels of PA-iron at one end of the field site [16]. From that field site in Changins, we regularly collected soil batches to study microbiome feedbacks on Arabidopsis (details in Table S4) and noticed that the strength of the feedbacks (measured as differential rosette area) also differed between soil batches (Fig. S13). Therefore, we reasoned that the levels of PA-iron might also explain the varying feedbacks on Arabidopsis growth. While the soil batches for Arabidopsis experiments were randomly collected without recording exact field coordinates, we had them analysed for their physicochemical soil parameters.

To probe this idea, we correlated the varying feedbacks on Arabidopsis growth with the different PA-iron concentrations in each soil batch. Consistent with the field work with wheat (*R* = −0.71, *p* < 0.001), we also found a significant negative correlation between the Arabidopsis growth feedbacks and PA-iron in soil (*R* = −0.5, *p* < 0.05; Fig. 5A). These correlations did not differ from each other (Fisher: z = 1.2; *p* > 0.05), suggesting that microbial feedbacks on wheat and Arabidopsis follow a similar relationship with soil PA-iron levels. Therefore, we saw support for the idea that soil PA-iron also predicts the varying strength of Arabidopsis growth feedbacks in the different soil batches.

Emerging from the wheat and Arabidopsis feedback findings, we hypothesised that increasing soil PA-iron levels would suppress the positive microbial feedbacks on plant growth. To test this, we conditioned Changins soil with wild-type and *bx1* maize plants at both high and low levels of iron fertilisation and subsequently scored the microbial feedbacks on Arabidopsis growth (Fig. 5B, upper part). Consistent with this hypothesis, Arabidopsis grew smaller on BX_minus_ compared to BX_plus_ soil in low iron conditions (*p* < 0.001) while high iron supplementation during soil conditioning eliminated this positive feedback and Arabidopsis grew equally on BX_minus_ and BX_plus_ soils (*p* > 0.05; Fig. 5B). Hence, with this iron supplementation experiment, we could experimentally validate the correlative evidence for an inverse relationship between soil PA-iron levels and microbial feedbacks on plant growth (wheat, Fig. 4B; Arabidopsis, Fig. 5A).

## Discussion

It is well established that (i) plants change their surrounding soil microbiome by the release of root exudates, (ii) such conditioned microbiomes impact the performance of a new plant generation and (iii) such ‘microbiome feedbacks’ depend much on the soil context. A key knowledge gap towards predictability is the identification of the underlying factors that explain the variation in strength and direction of soil microbiome feedbacks on plant growth [10, 50]. Here, we reported a new field site where local soil heterogeneity explained the variation in microbiome feedbacks on wheat height. We used this data combined with data of previous field experiments to identify the factor(s) that explain the variability of microbial feedbacks using multivariate, correlation and modelling analyses. Across multiple field sites and extended to Arabidopsis, we found that soil levels of PA-iron are strongly associated with the varying strength of the microbiome feedbacks. Below, we describe the emerging model and discuss its possible underlying mechanisms and the implications of soil heterogeneity.

### Model: positive feedbacks at low iron levels

**Fig. 6:**
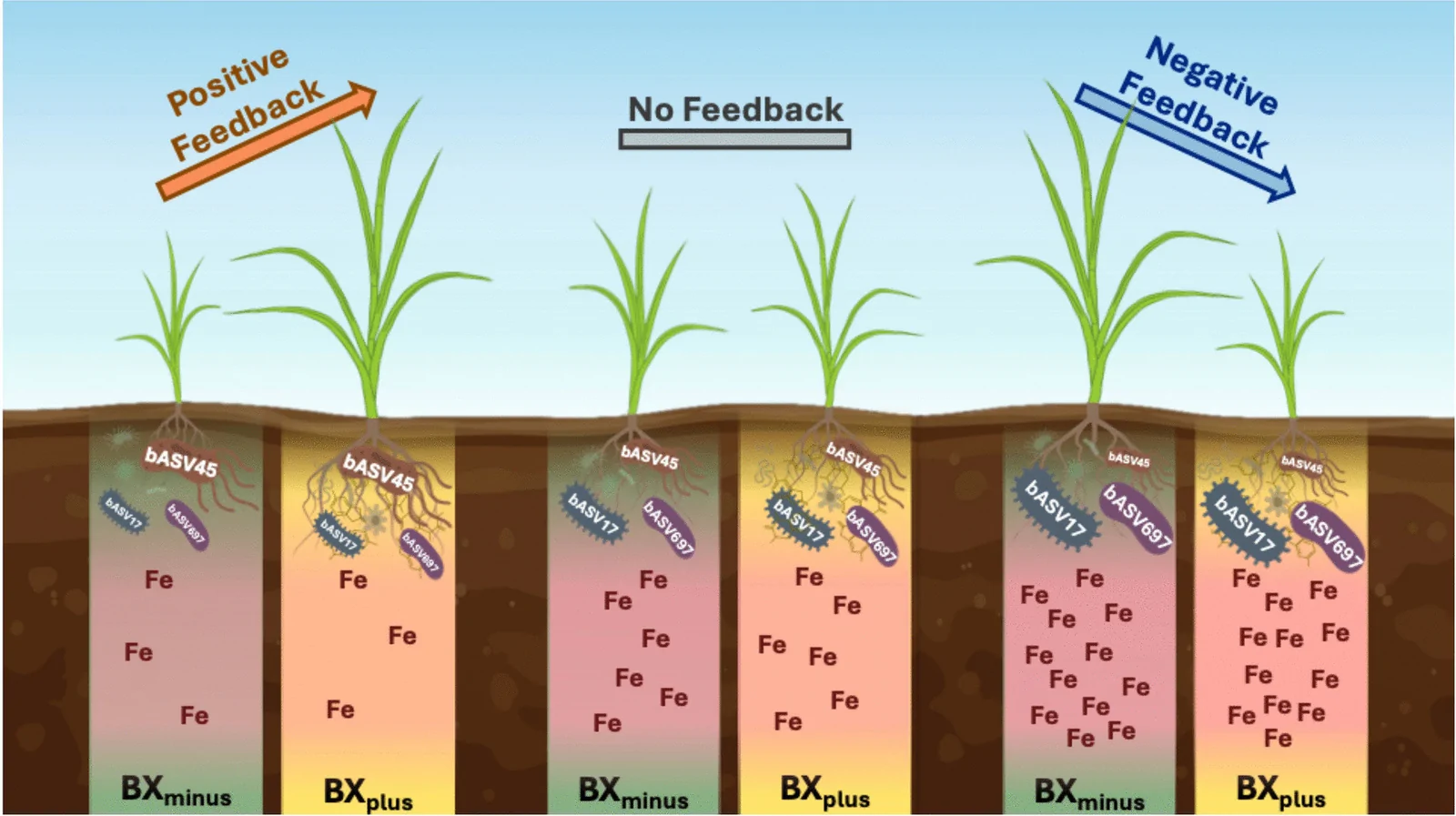
Dependency of BX-mediated feedbacks on plant-available iron in wheat. Model illustrating how local plant-soil feedbacks in wheat, growing on BX_minus_ or BX_plus_ conditioned soil, are influenced by plant-available iron concentrations and the microbiome. At low plant-available iron levels, wheat performs better on BX_plus_ conditioned soil than on BX_minus_ conditioned soil. As plant-available iron increases, this performance difference diminishes. At high plant-available iron concentrations, the direction of the feedback reverses, and wheat grown on BX_minus_ conditioned soil outperforms wheat grown on BX_plus_ conditioned soil. The same wheat feedback direction as observed for PA-iron was found for *Mesorhizobium* bASV697 and *Bacillus* bASV17 abundances, whereas *Pseudomonas* bASV45 showed the opposite pattern

Analysing the new field data from the Reckenholz site, alone as well as when combined with the Posieux and Changins sites, revealed consistency in two things: first, maize’s legacy microbiomes differed between wild-type and *bx1* plants (Figs. 2BC, 3DE) and second, the strength of their resulting feedbacks on wheat largely depended on the physicochemical soil parameters (Figs. 2D, 3F). PA-iron was the most important and most stable predictor to explain local height feedbacks of wheat (Fig. 4). We found this inverse relationship between soil iron and wheat feedbacks also to apply to Arabidopsis feedbacks where the strength varied with iron levels in the different soil batches collected from the Changins site (Fig. 5A). The following model emerges from taking all findings of this study together (Fig. 6): the microbial communities in BX_plus_ soils provoke positive feedbacks on wheat or Arabidopsis growth at low levels of PA-iron in soil while they cause no or even negative feedbacks at high iron levels. Testing this model with iron fertilisation revealed that the positive growth feedbacks of BX_plus_ microbiomes were lost at elevated soil iron levels (Fig. 5B). In conclusion, low levels of PA-iron link with beneficial feedbacks of the BX_plus_ microbiome on plant growth. In earlier work we have established the microbiome dependence of these feedbacks through sterilisation and microbiota complementation experiments [23, 47] and this study identifies iron as key player. The next frontier is now to examine the mechanistic linkage how iron connects to microbiome mediated feedbacks for instance by performing sterilisation and microbiota complementation experiments at different iron availabilities. Future work shall aim at identifying which microbes and how they promote plant growth under low-iron conditions.

### Bacterial predictors

Next to PA-iron as highly stable predictor, three bacterial predictors have been detected (Fig. S9B). These were sequences assigned to a *Bacillus* (bASV17), a *Pseudomonas* (bASV45) and a *Mesorhizobium* bacterium (bASV697). *Bacillus* bASV17 and *Mesorhizobium* bASV697 were negatively correlated to the feedback on plant height while *Pseudomonas* bASV45 showed a positive correlation (Figs. 4, 6). Previous studies support potential links between these genera, iron availability, and plant performance. For instance, many *Pseudomonas* strains like GRP3A, PRS9 or RSP5 are well-known siderophore producers [46]. Inoculation with these strains under low iron conditions has shown increased Fe accumulation, higher seed germination rates and more biomass in cereals, such as maize. Additionally, these strains were also antagonists against phytopathogens such as *Colletotrichum dematium*, *Rhizoctonia solani* and *Sclerotium rolfsii* [44, 46]*.* In contrast, *Mesorhizobium* species are primarily known for their nitrogen-fixing capabilities in legumes, and their role in iron mobilization in wheat remains less clear. A study by Verma et al. [52] reported that a co-inoculation of a *Mesorhizobium* sp. BHURC03, a *Pseudomonas aeruginosa* strain BHUPSB02 and a *Bacillus megaterium* BHUPSB14 showed significant enhancement in growth and nutrient acquisition, such as phosphorus and iron, in chickpeas. Further investigation on these bacterial predictors is required and ideally bacterial strains matching the predictive *Bacillus* (bASV17), *Pseudomonas* (bASV45) and *Mesorhizobium* (bASV697) could be isolated for functional assays at varying levels of PA-iron in soil.

### BXs as iron chelators

The above-mentioned model is challenged by the known function of BXs to act as phytosiderophores chelating iron for plant uptake [22]. Therefore, it appears plausible that feedback plants may benefit from residual BX-iron complexes in soil. Interestingly, it has indeed been demonstrated that non-BXs-producing plants, such as oat and rice, can benefit from BX-iron chelates from the surrounding environment [59]. Naïvely, this would explain that Arabidopsis, also a non-producing species, might benefit from BX-iron complexes under low PA-iron conditions. However, there is a very clear finding that contradicts such a plausible explanation: although not specifically quantifying BX-Fe complexes, we found very strong positive correlations between soil levels of PA-iron and both DIMBOA-Glu and DIMBOA levels in soil (Fig. S12). These two compounds are the most abundantly exuded BXs in maize [23] and are the BX species that actually chelate iron [22, 23]. The naïve explanation cannot work because the growth benefit is seen at low iron conditions, where the measured levels of soil DIMBOA-Glu and DIMBOA were also low. Taken together, it is unlikely that the phytosiderophore function of plant BXs would explain the growth benefit seen at low iron conditions. Instead, BXs might function to recruit a beneficial microbiome under low-iron conditions where possibly the plants benefit from either more growth-promoting microbes and/or microbes with dedicated siderophores. These latter possibilities require experimental testing e.g. by comparing bacterial isolates from BX_minus_ and BX_plus_ soils for their capacity to promote growth and to mobilise or solubilise PA-iron.

### Local soil conditions—essential co-variable in field studies

The dependency of microbial feedbacks on their soil environmental context is well recognised [10, 50]. While varying feedback responses are commonly found comparing different field soils, they are less obvious and require higher numbers of replicate plots to detect them in a fine-grained manner within fields. Each of our field experiment was conducted with 10 replicated strips (Posieux) or plots (Changins, Reckenholz) per soil conditioning. At Changins, where the field has a characteristic physicochemical gradient in soil, the BX-induced microbial feedbacks significantly changed along the gradient, while the high variation did not allow us to detect differences at field-scale [16]. Analogous at Reckenholz: wheat performance was unaffected (in statistical terms) when analysed at field-scale but significant differences were found between BX_minus_ and BX_plus_ soils when considering the local feedback (height, Fig. 1C; other phenotypes, Fig. S5). These examples highlight the importance of considering local soil heterogeneity for analysis and interpretation of field studies. In contrast to pot experiments, where field soil can be homogenised before filling, field studies require fine-grained analyses of the local soil parameters to account for spatial heterogeneity. We argue that the local soil physicochemical parameters present an essential co-variable for field studies to successfully disentangle the complex relationships between plant performance in response to the microbes in a given soil environment.

### Soil heterogeneity – essential for microbiome feedback experiments

Ironically, we had ignored the above conclusion for our experiments with Arabidopsis where we neglected local soil heterogeneity when sampling the different field soil batches. In the past, we did not pay attention *where* in the field we sampled soil for our pot experiments. Arabidopsis feedbacks were consistent within but inconsistent between different soil batches (Fig. S13, Table S4). Positive feedbacks were recorded for soil batches BS04, BS07 or BS09 while negative feedback was seen with the others. The strong relationship between soil PA-iron and feedback strength in wheat (Fig. 4, Gfeller et al. [16]) prompted us – fortunately we had all batches analysed for their physicochemical soil parameters – to examine PA-iron as possible driver of the varying feedbacks on Arabidopsis growth. Importantly, the significant negative relationship between Arabidopsis feedbacks and PA-iron (Fig. 5A) was consistent with wheat and could be validated with a fertilisation experiment. Using a soil batch with low iron levels, the positive growth feedback was lost upon enhancing soil iron levels (Fig. 5B). In retrospect, we learned the important lesson that local soil heterogeneity, not only as co-variable for field studies, is also essential for microbiome feedback experiments with natural soil under controlled conditions.

## Conclusions

The initial observation that microbiome feedbacks are highly dependent on the soil physicochemical parameters could be narrowed down to PA-iron as the main factor. The key finding of this study is that the levels of PA-iron in soil are associated with the strength of microbial feedbacks both on wheat and Arabidopsis. Low levels of PA-iron were needed for the beneficial soil microbiota (in this study, conditioned by BX-producing plants) to provoke positive growth feedbacks. In contrast, higher levels of PA-iron resulted in no- or even negative microbial feedbacks. An indirect conclusion of this finding is that fertilisation, i.e. enhancing PA-iron levels, is derogatory to beneficial microbial feedbacks. In simplified terms, when plants are nutritionally supported by fertilisers, they do not need or benefit from beneficial soil microbiomes. Thinking practically – e.g. about crop rotation at reduced fertiliser inputs – it will be important (i) to understand the environmental factors (e.g. low iron conditions) and (ii) to identify beneficial plant microbiomes (e.g. of BX producing maize plants) that then positively feedback on the next crop generation. It comes down to solving the context-dependency of microbial feedbacks, allowing then to take informed decisions for microbiome-assisted cropping practices that eventually enhance agricultural sustainability.

## Supplementary Information

Supplementary Material 1.

## Data Availability

The raw microbiome sequencing data is deposited at the European Nucleotide Archive ([http://www.ebi.ac.uk/ena]) under the study accessions PRJEB59165, PRJEB53704 and PRJEB94164. Datasets and code used in this study to analyse microbiome sequencing data, to do statistical analyses and to create figures are available on GitHub (For review: available as zip file, otherwise: [https://github.com/PMI-Basel/Waelchli/_et/_al/_Reckenholz/_field/_experiment]). The GitHub repository has two parts with “A” containing information and bash and R code for microbiome analysis as follows: A1) Metafile containing barcode, primer and sample assignment information A2) raw sequencing file names and A3) code to analyse microbiome sequencing data. The part “B” contains the raw data and R code for the following analyses: B01) soil chemistry, B02) statistic microbiome analysis, B03) BX soil concentrations, B04) wheat feedback, B05) local height feedback – soil chemistry interaction, B06) local height feedback – microbiome interaction, B07) modelling approach, B08) Arabidopsis feedback experiments.
