# Supplementary material for: Soil iron modulates beneficial maize microbiome feedbacks in rotations with wheat
Source: Microbiome. 2026 Aug 4;14:223. doi: 10.1186/s40168-026-02493-8 (PMC13628717; doi:10.1186/s40168-026-02493-8)
Supplement: Supplementary file 1 [file 40168_2026_2493_MOESM1_ESM.docx]

**Supplementary Information**

Title: Soil iron modulates beneficial maize microbiome feedbacks in rotations with wheat

Authors: Jan Waelchli, Henry Janse van Rensburg, Katja Stengele, Viola D’Adda, Selma Cadot, Veronica Caggìa, Valentin Gfeller, Klaus Schlaeppi

Index

- Supplementary figures
- Supplementary tables

| maize  wheat |  |
| --- | --- |

**Figure S1 | Experimental setup per field plot. (A)** For conditioning, maize was grown in strips with 75 cm distance. At the end of the conditioning phase, two soil samples and maize roots per plot were taken marked by red pins for physicochemical soil analysis and microbiota profiling. **(B)** During feedback phase wheat was grown in strips with 15 cm between them. Wheat height was measured from 10 randomly selected plants on the blue labelled line, *Oulema melanopus* larvae were counted from 10 randomly selected plants on the yellow line and the chlorophyll content were measured from 10 randomly selected plants on the green line. To measure the biomass, 1 meter of wheat was harvested, dried and weighted. The roots system of two wheat plant per plot were samples and used for microbiota profiling. After wheat has produced kernels, the core of each plot was harvested, and the kernel weight was determined.

**Figure S2 | Calculation of *local differences*.** This figure illustrates how *local differences* of wheat phenotypes were calculated. As an example, we show the calculation of local wheat height differences for the bx06 and WT03 plots at the Reckenholz field site. For BX_minus_ conditioned bx06 plots, the local wheat height difference was obtained by subtracting the mean height of the surrounding BX_plus_ conditioned plots from the plot’s wheat height. Conversely, for BX_plus_ conditioned WT03 plots, the mean height of the surrounding BX_minus_ conditioned plots was subtracted from the plot’s wheat height. The number of surrounding plots used to calculate the local feedback differences is indicated at the bottom left of each plot.

**Figure S3 | Rarefaction of maize and wheat root microbiome sequencing data.** We sequenced samples from the bacterial and fungal maize root communities at the end of the conditioning phase and the bacterial wheat root communities towards the end of the feedback phase. Samples were normalized by rarefaction to **(A)** 9’000 bacterial reads and **(B)** 1’000 fungal reads.

**Figure S4 | Maize phenotyping.** Maize biomass (as dry weight) was measured at the end of the conditioning phase. **(A)** No significant differences in biomass were observed between the wild type and the *bx1* mutant (t-test, replicates n=10). **(B)** Accounting for the local soil condition, by calculating *local* biomass differences (see Methods), revealed again no differences (t-test, replicates n=10).

**Figure S5 | Wheat phenotyping Reckenholz.** Wheat phenotypes were measured towards the end of the feedback phase. The plant height is shown in **Fig. 1C** while here we show **(A)** the measured chlorophyll content, **(B)** the dried plant biomass, **(C)** the number of *Oulema melanopus* larvae on the two newest tillers and **(D)** the kernel yield. For none of them we find a difference between wheat growing on BX_plus_ or BX_minus_ conditioned soil. Reducing the spatial variation by calculation local feedbacks (see methods) shows differences for **(E)** the local chlorophyll content differences and **(H)** the local kernel yield differences but not for **(F)** the local biomass differences and **(G)** the local insect infestation differences (t-test, p-value displayed in panel).

**Figure S6 | Wheat phenotyping across three field experiments.** Wheat phenotypes were measured towards the end of the feedback phase. For each field we show **(A)** the plant height, **(B)** the measured chlorophyll content, **(C)** the dried plant biomass, **(D)** the number of *Oulema melanopus* larvae on the two newest tillers and **(E)** the kernel yield. For none of them we find a difference for all three fields between wheat growing on BX_plus_ or BX_minus_ conditioned soil. Reducing the spatial variation by calculation local feedbacks (see methods) shows differences in all three fields for **(F)** the local height differences, but not for **(G)** the local chlorophyll content differences, **(H)** the local biomass differences, **(I)** the local insect infestation differences and **(J)** the local kernel yield differences (t-test, p-value displayed in panel).

**Figure S7 | Physicochemical soil properties from all maize-wheat plant-soil feedback experiments.** Maize-wheat plant soil-feedback experiments were performed in Changins, Posieux and Reckenholz. **(A)** PCA show Euclidean distances between measured physicochemical soil parameters at the end of the conditioning phase with the corresponding loadings indicating the contribution of physicochemical soil properties. **(B)** The quantities of each soil property are tested for differences among the different field sites with an Analysis of Variance (ANOVA). P-values are corrected for multiple testing by the Bonferroni-Holm method. Soil properties are plant-available nutrients (PA), partly plant-available nutrients (PPA), unavailable nutrients (UA), soil texture, humus content and pH. Colours and shapes represent field sites.

**Figure S8 | Collinearity of physicochemical soil properties from all maize-wheat plant-soil feedback experiments. (A)** Physicochemical soil parameters measured at the Changins, Posieux, and Reckenholz field sites were correlated with each other and are presented as a correlation matrix. **(B)** In addition, variance inflation factors (VIF) were calculated to assess collinearity among variables.

**Figure S9 | Three-Step Model Selection Approach.** **(A)** The local height feedback was modeled against 20 physicochemical soil properties and all bacterial and fungal ASVs with a minimum occurrence in at least 4 samples and a minimal total abundance of 0.15% (bacteria: 132 ASV, fungi: 56 ASVs). A least absolute shrinkage and selection operator (LASSO) regression approach was chosen with repeated subsample-based variable stability analysis. Step 1: For each of the 500 iterations a random seed was set, 80% of the samples were randomly selected as input and k-fold Cross-Validation was used to tune the λ-parameter. Step 2: Predictors with a lower selection frequency than 25% were considered as unstable and were discarded. **(B)** The plot shows the selection frequency of all predictors that have been identified by all LASSO regression with a frequency of at least 1%. Predictors were categorized by their selection stability: low (25–50%), moderate (50–75%) or high (75–100%).

**Figure S10 | Comparison of mixed-effects models for predicting local height feedback.** Mixed-effects models were fitted with local height feedback as the response variable and field site included as a random effect. Starting from the null model, predictors identified by the LASSO analysis were added either individually or all together. Model performance was evaluated using Akaike Information Criterion (AIC). The addition of each individual predictor increased significantly the model performance, but the best model performance was found by adding all four predictors. (χ² statistics and p-values are indicated above the bars).

**Figure S11 | Predictors in Wheat Root Microbiome.** With a LASSO modelling approach, bacterial ASVs has been identified to predict the local height feedback based on their abundances in the maize microbiome (**Fig. 4A**). Those abundances were significantly correlated with local height feedback (**Fig. 4B**). Here we test whether the abundances of the same ASVs in the wheat root microbiome were also correlated with local height feedback. Pearson's correlation coefficient (R) and Bonferroni-Holm adjusted p-value are labelled at the top. Colours of facet titles represent the direction of correlation between predictor and local height feedback.

 **Figure S12 | Predictors – Benzoxazinoids Correlation.** Benzoxazinoid-concentration has been measured on all BX_plus_ conditioned plots in Changins and Posieux, but not in Reckenholz. Selected predictors were correlated with the 3 most exuded BX. Pearson's correlation coefficient (R) and Bonferroni-Holm adjusted p-value are labelled at the top. Significant p-values are marked in red. Shapes represent field sites. Colours of vertical facet titles represent the direction of correlation between predictor and local height feedback.

**Figure S13 | Arabidopsis: variation in feedback magnitude.** Rosette feedback of Arabidopsis on BX_plus_ or BX_minus_ conditioned soils from different soil batches. Data were z-score normalised per experiment. Points represent mean differences ± standard errors. Positive values indicate Arabidopsis growing bigger on BX_plus_ conditioning soil while Arabidopsis with negative values grow better on BX_minus_ conditioned soil. For details on the calculation of rosette feedack, refer to the Materials and Methods section. A t-test per replicate was performed to determine differences between feedbacks on BX_plus_ and BX_minus_ conditioned soils. Asterisks mark significances (*<0.05, **<0.01, ***<0.001).

**Table S1: Number of replicates**

Number of used replicates for all measured values on the three field sites during the conditioning and feedback phase.

| **Number of Replicates** | | | | | | |
| --- | --- | --- | --- | --- | --- | --- |
| **Location** | **Changins** | | **Posieux** | | **Reckenholz** | |
| **Conditioning** | **BX_plus_** | **BX_minus_** | **BX_plus_** | **BX_minus_** | **BX_plus_** | **BX_minus_** |
| **Maize Biomass** | - | - | -^1^ | -^1^ | 10 | 10 |
| **Wheat Height** | 10 | 10 | 10 | 10 | 10 | 10 |
| **Wheat Chlorophyll** | 10 | 10 | 10 | 10 | 10 | 10 |
| **Wheat Biomass** | 10 | 10 | 10 | 10 | 10 | 10 |
| **Wheat Insect Infestation** | 10 | 10 | 10 | 10 | 10 | 10 |
| **Wheat Kernel Yield** | 10 | 10 | 10 | 10 | 10 | 10 |
| **Physicochemical Soil Parameters** | 10 | 10 | 4^2^ | 4^2^ | 7^3^ | 8^3^ |
| **Maize Root Bacteria** | 10 | 10 | 8^4^ | 9^4^ | 10 | 9^4^ |
| **Maize Root Fungi** | 10 | 9^4^ | 9^4^ | 10 | 10 | 10 |
| **Modelling** | 10 | 9^5^ | 2^5,6^ | 4^5,6^ | 7^5^ | 7^5^ |
| **Wheat Root Bacteria** | 10 | 10 | 9^4^ | 10 | 9^4^ | 9^4^ |

^1^Samples were collected but not used here ^2^Samples were pooled for soil analysis (see methods)
^3^Samples had not enough collected soil for soil analysis
^4^Samples removed due to too low sequencing depth or detected as outlier (CLOUD method)
^5^Samples removed with missing soil analysis, maize root bacteria or maize root fungi sample
^6^Samples were pooled as for soil analysis (see methods)

**Table S2: Correlations Reckenholz**

Correlation analysis of wheat’s local phenotype feedback (height, chlorophyll, biomass, insect infestation and kernel yield) from Reckenholz to all principal component and coordinate axes of the soil physicochemical properties and microbial community data explaining at least 10% of variation (Values were derived from PCA and PCoA, respectively). The correlation coefficient rho (R-value) and p-value and were calculated with a Pearson correlation test. The axes with significant correlations are marked in bold.

| **Reckenholz field site** | | | |
| --- | --- | --- | --- |
| **Correlation Local Height Feedback to** | **% of Variation** | **R value** | **p-value** |
| **Physicochemical Soil Chemistry PC1** | **33.58%** | **-0.741** | **0.0016** |
| Physicochemical Soil Chemistry PC2 | 23.55% | -0.412 | 0.1272 |
| Physicochemical Soil Chemistry PC3 | 13.10% | -0.063 | 0.8238 |
| Root Bacteria PCo1 | 71.82% | -0.297 | 0.2166 |
| Root Bacteria PCo2 | 19.46% | 0.102 | 0.6790 |
| Root Fungi PCo1 | 45.65% | -0.094 | 0.6927 |
| Root Fungi PCo2 | 24.36% | -0.060 | 0.8010 |
| Root Fungi PCo3 | 14.53% | -0.069 | 0.7732 |
| **Correlation Local Chlorophyll Feedback to** | **% of Variation** | **R value** | **p-value** |
| Physicochemical Soil Chemistry PC1 | 33.58% | -0.304 | 0.3373 |
| Physicochemical Soil Chemistry PC2 | 23.55% | 0.285 | 0.3684 |
| Physicochemical Soil Chemistry PC3 | 13.10% | 0.245 | 0.4412 |
| Root Bacteria PCo1 | 71.82% | -0.220 | 0.3645 |
| Root Bacteria PCo2 | 19.46% | -0.303 | 0.2077 |
| Root Fungi PCo1 | 45.65% | -0.017 | 0.9427 |
| Root Fungi PCo2 | 24.36% | -0.219 | 0.3542 |
| Root Fungi PCo3 | 14.53% | -0.363 | 0.1157 |
| **Correlation Local Biomass Feedback to** | **% of Variation** | **R value** | **p-value** |
| Physicochemical Soil Chemistry PC1 | 33.58% | -0.283 | 0.3063 |
| Physicochemical Soil Chemistry PC2 | 23.55% | -0.283 | 0.3064 |
| Physicochemical Soil Chemistry PC3 | 13.10% | 0.366 | 0.1800 |
| Root Bacteria PCo1 | 71.82% | 0.168 | 0.4916 |
| Root Bacteria PCo2 | 19.46% | -0.321 | 0.1804 |
| Root Fungi PCo1 | 45.65% | -0.126 | 0.5975 |
| Root Fungi PCo2 | 24.36% | -0.420 | 0.0655 |
| Root Fungi PCo3 | 14.53% | 0.002 | 0.9921 |
| **Correlation Local Insect Infestation Feedback to** | **% of Variation** | **R value** | **p-value** |
| Physicochemical Soil Chemistry PC1 | 33.58% | 0.357 | 0.1918 |
| Physicochemical Soil Chemistry PC2 | 23.55% | -0.202 | 0.4709 |
| Physicochemical Soil Chemistry PC3 | 13.10% | -0.257 | 0.3552 |
| Root Bacteria PCo1 | 71.82% | -0.013 | 0.9568 |
| Root Bacteria PCo2 | 19.46% | -0.300 | 0.2118 |
| Root Fungi PCo1 | 45.65% | -0.180 | 0.4483 |
| Root Fungi PCo2 | 24.36% | -0.327 | 0.1592 |
| Root Fungi PCo3 | 14.53% | 0.183 | 0.4409 |
| **Correlation Local Kernel Yield Feedback to** | **% of Variation** | **R value** | **p-value** |
| Physicochemical Soil Chemistry PC1 | 33.58% | -0.504 | 0.0553 |
| Physicochemical Soil Chemistry PC2 | 23.55% | 0.268 | 0.3350 |
| Physicochemical Soil Chemistry PC3 | 13.10% | 0.223 | 0.4237 |
| Root Bacteria PCo1 | 71.82% | -0.310 | 0.1969 |
| Root Bacteria PCo2 | 19.46% | -0.375 | 0.1135 |
| Root Fungi PCo1 | 45.65% | 0.213 | 0.3677 |
| Root Fungi PCo2 | 24.36% | -0.130 | 0.5836 |
| Root Fungi PCo3 | 14.53% | -0.019 | 0.9356 |

**Table S3: Correlations all field sites**

Correlation analysis of wheat’s local phenotype feedback (height, chlorophyll, biomass, insect infestation and kernel yield) from Reckenholz to all principal component and coordinate axes of the soil physicochemical properties and microbial community data explaining at least 10% of variation (Values were derived from PCA and PCoA, respectively). The correlation coefficient rho (R-value) and p-value and were calculated with a Pearson correlation test. The axes with significant correlations are marked in bold.

| **All field sites** | | | |
| --- | --- | --- | --- |
| **Correlation Local Height Feedback to** | **% of Variation** | **R value** | **p-value** |
| Physicochemical Soil Chemistry PC1 | 38.37% | 0.177 | 0.2559 |
| **Physicochemical Soil Chemistry PC2** | **25.06%** | **-0.597** | **2.40E-05** |
| **Physicochemical Soil Chemistry PC3** | **16.56%** | **0.317** | **0.0381** |
| **Root Bacteria PCo1** | **57.81%** | **0.423** | **0.0031** |
| Root Bacteria PCo2 | 23.76% | -0.118 | 0.4279 |
| Root Fungi PCo1  Root Fungi PCo2 | 68.24%  18.08% | 0.272  -0.186 | 0.0589  0.2006 |
| **Correlation Local Chlorophyll Feedback to** | **% of Variation** | **R value** | **p-value** |
| **Physicochemical Soil Chemistry PC1** | **38.37%** | **-0.379** | **9.58E-09** |
| Physicochemical Soil Chemistry PC2 | 25.06% | -0.067 | 0.3310 |
| **Physicochemical Soil Chemistry PC3** | **16.56%** | **0.397** | **1.60E-09** |
| Root Bacteria PCo1 | 57.81% | -0.275 | 0.0610 |
| Root Bacteria PCo2 | 23.76% | 0.225 | 0.1281 |
| **Root Fungi PCo1**  Root Fungi PCo2 | **68.24%**  18.08% | **-0.403**  0.050 | **0.0040**  0.7349 |
| **Correlation Local Biomass Feedback to** | **% of Variation** | **R value** | **p-value** |
| **Physicochemical Soil Chemistry PC1** | **38.37%** | **-0.312** | **3.00E-06** |
| **Physicochemical Soil Chemistry PC2** | **25.06%** | **-0.362** | **4.47E-08** |
| **Physicochemical Soil Chemistry PC3** | **16.56%** | **-0.307** | **4.49E-06** |
| Root Bacteria PCo1 | 57.81% | 0.151 | 0.3105 |
| Root Bacteria PCo2 | 23.76% | -0.146 | 0.3260 |
| Root Fungi PCo1  Root Fungi PCo2 | 68.24%  18.08% | -0.137  0.213 | 0.3485  0.1414 |
| **Correlation Local Insect Infestation Feedback to** | **% of Variation** | **R value** | **p-value** |
| **Physicochemical Soil Chemistry PC1** | **38.37%** | **0.237** | **0.0004** |
| **Physicochemical Soil Chemistry PC2** | **25.06%** | **0.296** | **1.03E-05** |
| **Physicochemical Soil Chemistry PC3** | **16.56%** | **-0.191** | **0.0049** |
| Root Bacteria PCo1 | 57.81% | 0.009 | 0.9523 |
| Root Bacteria PCo2 | 23.76% | 0.021 | 0.8908 |
| Root Fungi PCo1  Root Fungi PCo2 | 68.24%  18.08% | 0.040  0.171 | 0.7847  0.2396 |
| **Correlation Local Kernel Yield Feedback to** | **% of Variation** | **R value** | **p-value** |
| **Physicochemical Soil Chemistry PC1** | **38.37%** | **-0.419** | **1.53E-10** |
| Physicochemical Soil Chemistry PC2 | 25.06% | 0.079 | 0.2485 |
| **Physicochemical Soil Chemistry PC3** | **16.56%** | **0.415** | **2.21E-10** |
| Root Bacteria PCo1 | 57.81% | -0.202 | 0.1733 |
| **Root Bacteria PCo2** | **23.76%** | **0.360** | **0.0128** |
| **Root Fungi PCo1**  Root Fungi PCo2 | **68.24%**  18.08% | **-0.422**  -0.058 | **0.0024**  0.6902 |

**Table S4: Collected soil batches to study microbiome feedbacks on Arabidopsis**

Multiple soil batches were collected on the field site in Changins. The soil was conditioned with WT and *bx1* maize and the feedbacks on Arabidopsis were measured.

| **Arabidopsis Datasets** | | | | | | | | |
| --- | --- | --- | --- | --- | --- | --- | --- | --- |
|  | | | **Conditioning Phase (Maize)** | | | **Feedback Phase (At)** | | |
| **Batch** | **Nr of Exp** | **Availability** | **Growth (w)** | **Type** | **Fertilization** | **Fertilization (d)** | **Growth (d)** | **Type** |
| BS04 | 2 | (Janse van Rensburg et al., 2025) | 12 | pot | weekly  (4w low^1^/8w high^2^) | 14^3^, 21^3^ | 35 | pot |
| BS04 | 1 | this study | 12 | pot | weekly  (4w low^1^/8w high^2^) | 23^4^ | 40 | pot |
| BS06 | 1 | this study | 12 | pot | weekly  (4w low^1^/8w high^2^) | 15^4^, 22^4^ | 49 | pot |
| BS07 | 1 | (Janse van Rensburg et al., 2025) | 12 | pot | weekly  (4w low^1^/8w high^2^) | 14^3^, 21^3^ | 35 | pot |
| BS07 | 1 | (Stengele et al., 2024) | 12 | pot | weekly  (4w low^1^/8w high^2^) | - | 42 | pot |
| BS08 | 1 | this study | 12 | pot | weekly  (4w low^1^/8w high^2^) | 14^3^, 21^3^ | 35 | pot |
| BS08 | 1 | this study | 12 | pot | weekly  (4w low^1^/8w high^2^) | 23^4^, 37^4^ | 42 | pot |
| BS09_high | 1 | (Stengele et al., 2024) | 12 | pot | weekly  (4w low^1^/8w high^2^) | 14^3^, 21^3^ | 35 | pot |
| BS09_low | 1 | (Stengele et al., 2024) | 12 | pot | weekly  (12w low^1^) | 14^3^, 21^3^ | 35 | pot |
| ChanNN | 1 | this study | 12 | field | conventional farming practice | 15^4^, 22^4^ | AUC^5^ | pot |
| ChanNS | 1 | this study | 12 | field | conventional farming practice | 15^4^, 22^4^ | AUC^5^ | pot |
| ChanSN | 1 | this study | 12 | field | conventional farming practice | 15^4^, 22^4^ | AUC^5^ | pot |
| ChanSS | 1 | this study | 12 | field | conventional farming practice | 15^4^, 22^4^ | AUC^5^ | pot |

^1^ Low iron: 100 mL of 0.2% Plantaaktiv Typ K, 0.001% Sequestrene Rapid
^2^ High iron: 200 mL of 0.2% Plantaaktiv Typ K, 0.02% Sequestrene Rapid. All was applied once a week.
^3^ Fertilization by watering with 10mL of 1/3 half strength Hoagland solution diluted in tap water
^4^ Fertilization by watering with 5mL undiluted half strength Hoagland solution
^5^AUC stands for the area under the curve of weekly rosette area measurements from 3 to 8 weeks of growth
